# Correction: Toader et al. Cognitive Crescendo: How Music Shapes the Brain’s Structure and Function. *Brain Sci.* 2023, *13*, 1390

**DOI:** 10.3390/brainsci14040365

**Published:** 2024-04-09

**Authors:** Corneliu Toader, Calin Petru Tataru, Ioan-Alexandru Florian, Razvan-Adrian Covache-Busuioc, Bogdan-Gabriel Bratu, Luca Andrei Glavan, Andrei Bordeianu, David-Ioan Dumitrascu, Alexandru Vlad Ciurea

**Affiliations:** 1 Department of Neurosurgery, “Carol Davila” University of Medicine and Pharmacy, 020021 Bucharest, Romania; corneliu.toader@umfcd.ro (C.T.); bogdan.bratu@stud.umfcd.ro (B.-G.B.); luca-andrei.glavan0720@stud.umfcd.ro (L.A.G.); andrei.bordeianu@stud.umfcd.ro (A.B.); david-ioan.dumitrascu0720@stud.umfcd.ro (D.-I.D.); prof.avciurea@gmail.com (A.V.C.); 2 Department of Vascular Neurosurgery, National Institute of Neurology and Neurovascular Diseases, 077160 Bucharest, Romania; 3 Department of Opthamology, “Carol Davila” University of Medicine and Pharmacy, 020021 Bucharest, Romania; 4 Central Military Emergency Hospital “Dr. Carol Davila”, 010825 Bucharest, Romania; 5 Department of Neurosciences, “Iuliu Hatieganu” University of Medicine and Pharmacy, 400012 Cluj-Napoca, Romania; 6 Neurosurgery Department, Sanador Clinical Hospital, 010991 Bucharest, Romania

## Figure Legend

In the original publication [1], there was a mistake in the legend for *Figure 4*. The legend was incomplete. The correct legend appears below.

**Figure 4.** This image depicts examples of the main possibilities of clinical therapies using music. The given context is music therapies and daily music listening in various situations, such as in groups or individually and active or passive listening. Music offers multiple cognitive advantages and might be perceived in multiple ways which are described as “capacities”. Underlying mechanisms of music processing were aforementioned in this study, audio-motor functions and neuroplasticity being of high interest. Multiple behavioral-cognitive benefits, as well as motricity and psychological status, are highly improved [61,104]. Preprinted from Brancatisano, Baird, and Thompson, 2020 [61], with permission from the authors.

The authors state that the scientific conclusions are unaffected. This correction was approved by the Academic Editor. The original publication has also been updated.

## References

[1] Toader C., Tataru C.P., Florian I.-A., Covache-Busuioc R.-A., Bratu B.-G., Glavan L.A., Bordeianu A., Dumitrascu D.-I., Ciurea A.V. (2023). Cognitive Crescendo: How Music Shapes the Brain’s Structure and Function. Brain Sci..

